# Preliminary study on the optical diagnosis of orbital rhabdomyosarcoma by Raman spectroscopy

**DOI:** 10.1038/s41598-024-60520-w

**Published:** 2024-04-28

**Authors:** Ling Jin, Nengli Dai, Xiaobo Yang

**Affiliations:** 1 Department of Ophthalmology, Union Hospital, Tongji Medical College, Huazhong University of Science and Technology, Wuhan, 430022 China; 2 Wuhan National Laboratory for Optoelectronics, Huazhong University of Science and Technology, Wuhan, 430074 China

**Keywords:** Biological techniques, Cancer, Medical research, Optics and photonics

## Abstract

To investigate the Raman spectral features of orbital rhabdomyosarcoma (ORMS) tissue and normal orbital tissue in vitro, and to explore the feasibility of Raman spectroscopy for the optical diagnosis of ORMS. 23 specimens of ORMS and 27 specimens of normal orbital tissue were obtained from resection surgery and measured in vitro using Raman spectroscopy coupled to a fiber optic probe. The important spectral differences between the tissue categories were exploited for tissue classification with the multivariate statistical techniques of principal component analysis (PCA) and linear discriminant analysis (LDA). Compared to normal tissue, the Raman peak intensities located at 1450 and 1655 cm^−1^ were significantly lower for ORMS (*p* < 0.05), while the peak intensities located at 721, 758, 1002, 1088, 1156, 1206, 1340, 1526 cm^−1^ were significantly higher (*p* < 0.05). Raman spectra differences between normal tissue and ORMS could be attributed to the changes in the relative amounts of biochemical components, such as nucleic acids, tryptophan, phenylalanine, carotenoid and lipids. The Raman spectroscopy technique together with PCA-LDA modeling provides a diagnostic accuracy of 90.0%, sensitivity of 91.3%, and specificity of 88.9% for ORMS identification. Significant differences in Raman peak intensities exist between normal orbital tissue and ORMS. This work demonstrated for the first time that the Raman spectroscopy associated with PCA-LDA diagnostic algorithms has promising potential for accurate, rapid and noninvasive optical diagnosis of ORMS at the molecular level.

Rhabdomyosarcoma is the most common mesenchymal tumor of childhood. Common primary sites include the head and neck, trunk, and extremities. Orbital rhabdomyosarcoma (ORMS) is the most common primary malignant orbital tumor of childhood, and its incidence rate is higher than the sum of other orbital sarcomas^1,2^. Due to the aggressiveness of the tumor, the 5-year survival rate for patients with ORMS in China is only about 55.7%^3^, even under treatments combining surgery, radiotherapy and chemotherapy. Early diagnosis and complete resection of the tumors are keys to improving the survival rate of patients with ORMS^4^.

To date, diagnosis of ORMS mainly relies on imageological examinations such as computed tomography (CT) and magnetic resonance imaging (MRI). While these traditional medical imaging modalities characterize the locations and anatomical relationships of the tumor, most of them have limitations in terms of pathological clues and molecular characterization^5^. Consequently, direct sampling for histological and molecular characterization is required for diagnosis and treatment planning. Pathological examination is still the gold standard for confirming the diagnosis. However, pathological diagnosis is subjective and wide ranges of inter-observer variation have been reported, especially for precancerous lesions and early-stage cancers. Furthermore, pathological examination is invasive and time-consuming, making it impossible for real-time diagnosis and difficult to provide immediate guidance for tumor resection surgery. Therefore, there is an urgent need to develop an accurate, noninvasive, real-time diagnostic technique for ORMS.

Raman spectroscopy is an optical vibrational technique that gives spectral tissue characteristics based on molecular signatures resulting from inelastic scattering of incident light. With the advances in laser and detector technology, and multivariate spectral analysis, Raman spectroscopy has the potential to detect molecular changes associated with tissue pathology and enable noninvasive, real-time optical diagnosis. Compared with current imaging techniques (eg, CT and MRI) and pathological examination, Raman spectroscopy provides biochemical information about different tissues, but may be more suitable for routine use because it is faster, less complicated, and relatively low cost. The use of Raman spectroscopy for differentiating premalignant and malignant lesions from normal tissue has been studied for various body sites^6–8^. However, until recently, the investigation of Raman spectroscopy for orbital tumor diagnosis is rarely reported.

Here, we presented a study in which Raman spectroscopy was used to discriminate samples of ORMS from normal orbital tissue in vitro, by developing a spectral model to extract most of the spectral features of orbital tissues using principal component analysis (PCA) and linear discriminant analysis (LDA). The results indicate that the technique has the potential to be applied to accurately diagnose ORMS in real-time in a non-destructive manner at the molecular level.

## Experimental materials and methods

### Specimen collection

The protocol for this study was approved by the Medical Ethics Committee of Tongji Medical College, Huazhong University of Science and Technology. All procedures conformed to the Declaration of Helsinki. Informed consents were obtained from all subjects or their legal guardians. Tissue specimens, including 23 ORMS tissues and 27 orbital normal tissues 5 mm away from the tumor, obtained during the resection procedure. Upon removal, the specimens were rinsed with sterile saline to remove the surface bloodstains and other contaminants and then were cut into a size of about 0.5*0.5 cm. The samples were immediately placed in a freezing tube for packaging and then stored at −80 °C until further use. The frozen samples were defrosted before detected by Raman spectroscopy. Following spectroscopic measurements, all specimens were fixed in 10% buffered formalin and sent for histopathologic evaluation.

### Spectral measurement and data processing

The experiment used a LabRAM HR800 Raman spectrometer from Horiba Jobin Yvon, France. The excitation power was set to 50mW. Raman scattered light was collected in the wavenumber interval from 600 to 1800 cm^−1^ in the molecular fingerprint region with a spectral resolution of approximately 1 cm^−1^. A hand-held fiber optic probe was connected to a 785 nm excitation laser and a high-resolution charge-coupled device spectroscopic detector. The probe consisted of a central excitation fiber optic (diameter 400 μm) surrounded by seven collection fibers (diameter 200 μm) that collected Raman scattering and transferred it to the spectrometer. The specimen was placed onto an aluminum sample holder through which the tip of the Raman probe exited. The probe was placed in direct contact with the samples for each measurement, with a total acquisition time of 10 s per measurement. The system was used to measure the Raman spectra at 4 to 6 various sites for each specimen. Multiple Raman spectra (~ 15–20) for each tissue site were measured. The mean spectrum is the average of overall Raman measurements taken from the specimen.

The raw spectra acquired from orbital tissues represented a combination of intense tissue autofluorescence, weak tissue Raman scattering signals and noise. Thus, the raw spectra were preprocessed by a first-order Savitsky–Golay filter to reduce noise. Due to the simplicity and effectiveness, an iterative fifth-order polynomial fitting was used to subtract the broad autofluorescence background in the noise-smoothed spectrum, and this polynomial was then extracted from the raw spectrum to yield the tissue Raman spectrum alone. Each of background-subtracted Raman spectra was then normalized to the integrated area under the curve from 600 to 1800 cm^−1^ to correct for variations in absolute spectral intensity and enable comparison of relative intensities^9^. A Student’s t-test was performed at each wavenumber to compare the mean spectral intensities between different groups within the data set with SPSS 20.0, and *p* < 0.05 was considered statistically significant.

The simplification and dimension reduction of spectral data were processed by PCA in SPSS software, and the principal components (PCs) with characteristic values (eigenvalues greater than 1) were extracted. As a second step, the statistically significant PCs were input into LDA and a tissue classification model was established to test the accuracy, sensitivity and specificity of Raman spectroscopy for the optical diagnosis of ORMS.

## Results

**Figure 1:**
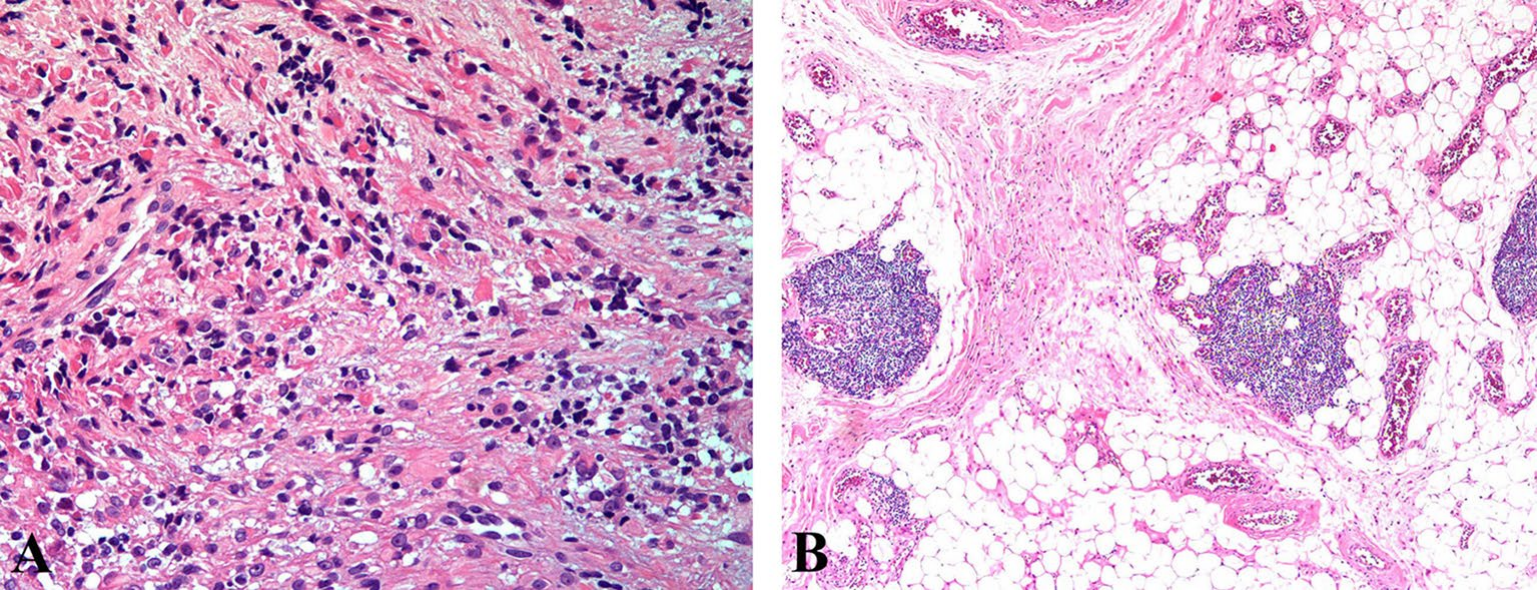
Representative hematoxylin and eosin (H&E)-stained histopathologic slides of embryonal ORMS tissue (**A**) and normal orbital tissue (**B**) (magnification: × 200). (**A**) Note the fascicles of pleomorphic, spindle-shaped cells with hyperchromatic nuclei and the eosinophilic myoblasts in ORMS. (**B**) The normal orbital tissue is predominately composed of fat, fibrous connective tissue and blood vessels.

**Figure 2:**
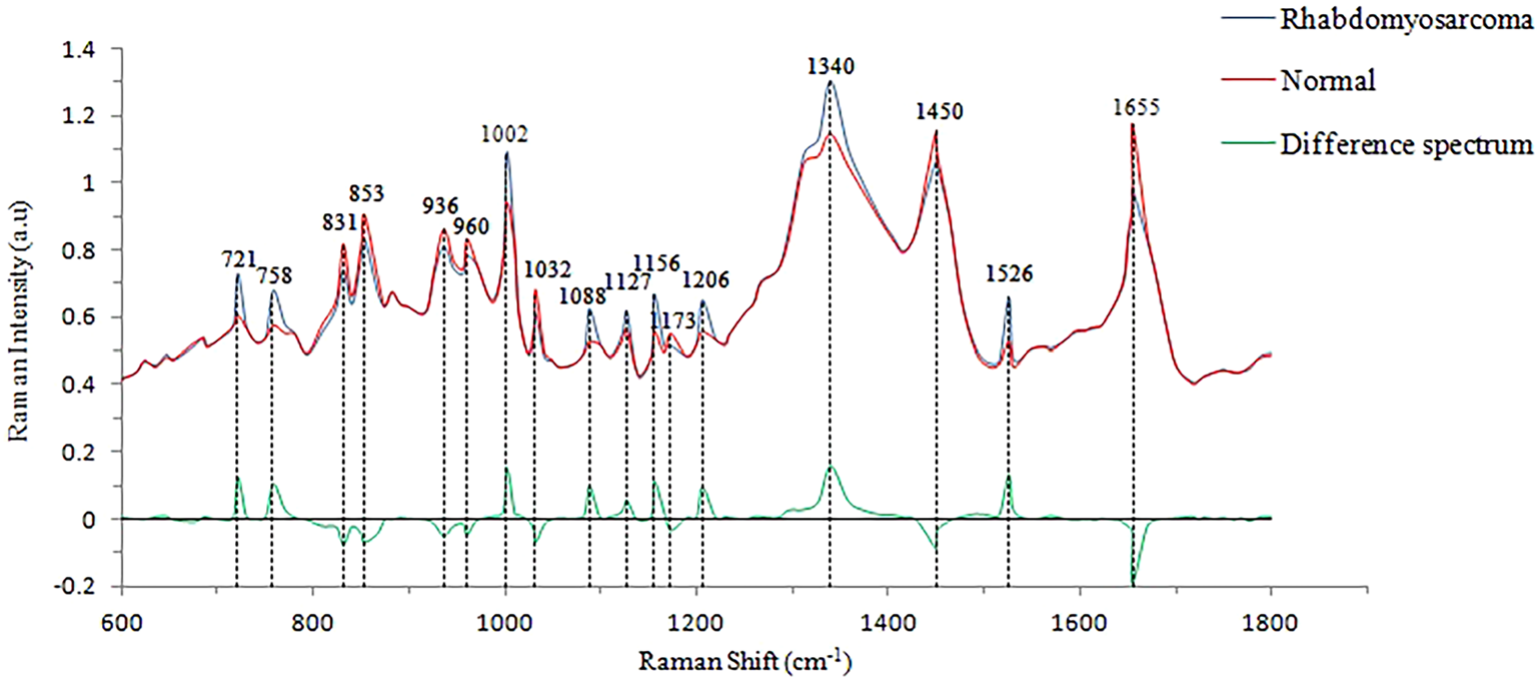
Mean Raman spectra of orbital rhabdomyosarcoma (ORMS) tissue (blue line, n = 23) and normal orbital tissue (red line, n = 27) in vitro. ORMS minus normal orbital tissue is used to represent the mean difference spectrum (green line), which is shown at the bottom.

While histopathology (Fig. 1) identifies the presence of the fascicles of pleomorphic, spindle-shaped cells with hyperchromatic nuclei as well as the eosinophilic myoblasts in embryonal ORMS, the fiberoptic Raman spectroscopy uncovers the biochemical and biomolecular changes occurring during carcinogenesis. The mean Raman spectra of ORMS and normal orbital tissue are shown in Fig. 2, in which a difference spectrum (ORMS minus normal tissue) was calculated to clarify the difference between the two spectra. The assignments of the highlighted spectral peaks referenced in the recent literature^10–13^are presented in Table 1.

**Table 1:** The peak positions and assignment of Raman spectra.

Raman peaks (cm^−1^)	Assignment
721	C-N band in nucleic acid
758	aromatic ring of tryptophan
831	DNA (PO_2_ asymmetric stretching), tyrosine
853	ν(C–C) of proline
936	ν(C–C) in α-conformation of proline
960	sterol ring stretch of cholesterol
1002	νs(C–C) ring breathing of phenylalanine
1032	ν(C–C) of phenylalanine
1088	nucleic acids backbone (PO_2_ symmetric stretching)
1127	ν(C–C) skeletal of acyl backbone in lipid
1156	ν(C=C) stretching mode in carotenoid
1173	C–C stretching of cholesterol, fatty acids—HO
1206	ν(C–C_6_H_5_) of tryptophan and phenylalanine
1340	δ(CH_2_CH_3_) of proteins and nucleic acids
1450	δ(CH_2_) of phospholipids and collagen
1526	ν(–C=C–) of carotenoid
1655	C=C stretching of lipids (unsaturated fatty acids)

**Figure 3:**
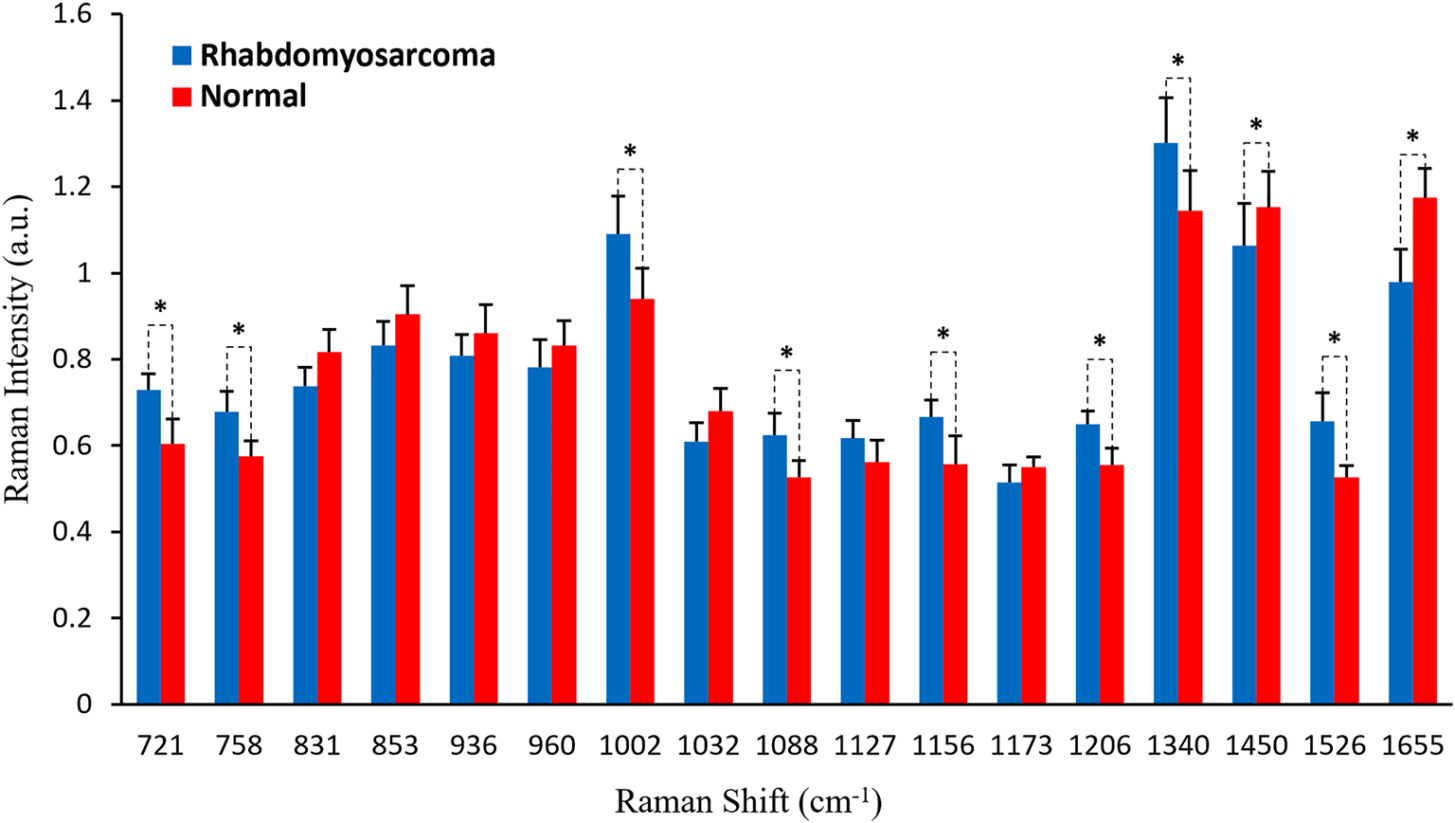
Comparison of peak intensities and standard deviations of Raman characteristic peaks between ORMS and normal tissue. *Note*: (*) indicates a significant difference for discriminating OMRS from normal tissue (*p* < 0.05).

As shown in Fig. 2, prominent Raman peaks are identified at 721, 758, 831, 853, 936, 960, 1002, 1032, 1088, 1127, 1156, 1173, 1206, 1340, 1450, 1526, 1655 cm^−1^ in both ORMS and normal orbital tissue. Figure 3 shows the intensity and standard deviation of the above all Raman peaks in the fingerprint region. Compared to normal tissue, the peak intensities located at 1450 and 1655 cm^−1^ were significantly lower for ORMS, while the peak intensities located at 721, 758, 1002, 1088, 1156, 1206, 1340, 1526 cm^−1^ were significantly higher (*p* < 0.05, unpaired 2-sided Student’s t-test).

**Figure 4:**
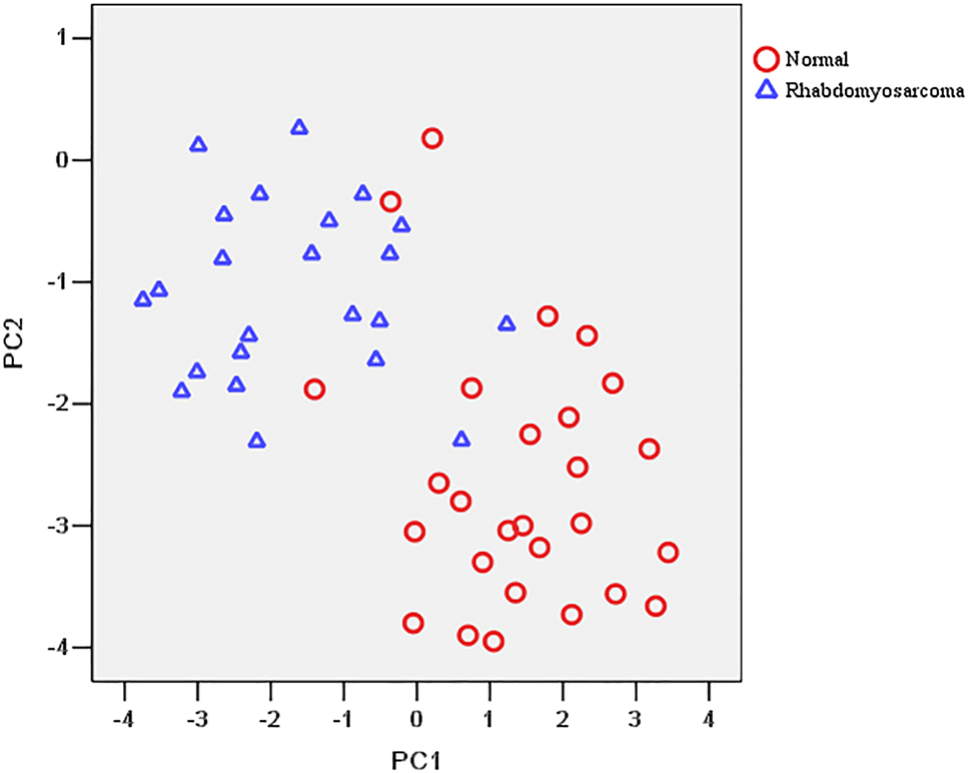
Scatter plots of the two diagnostically significant PC scores (PC1 versus PC2) for rhabdomyosarcoma and normal tissue Raman spectra.

**Figure 5:**
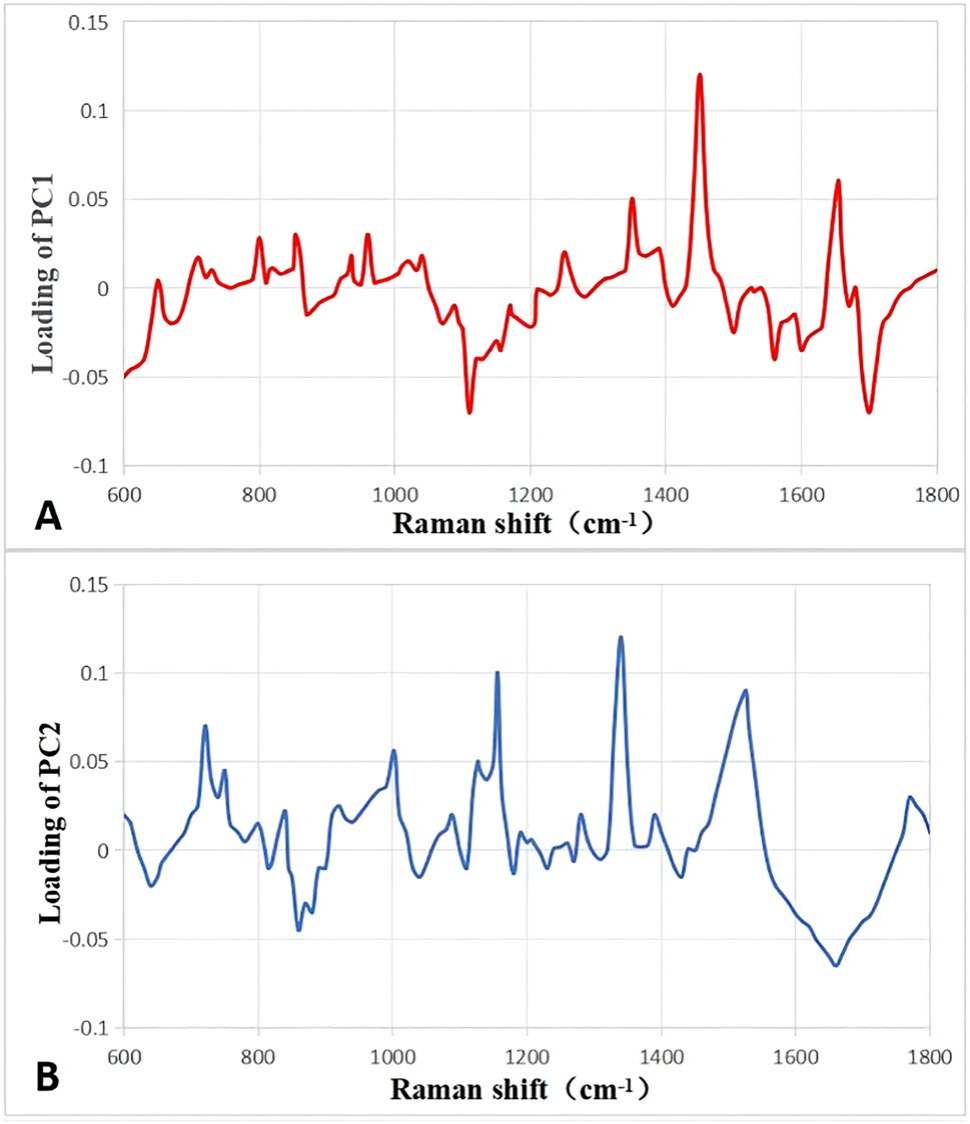
PCA loadings plots for PC1 (**A**) and PC2 (**B**).

The Raman data were then processed using PCA. The two most significant PCs (PC1 and PC2) that allowed us to differentiate ORMS group from normal orbital tissue group were selected. The scatter plots are shown in Fig. 4, displaying that the two groups of tissue spectra can be well separated using PC1 and PC2. To determine which Raman features are responsible for the discrimination, the loadings plot for PC 1 and PC2 are depicted in Fig. 5. In case of loading PC1, positive peaks have been observed at 1450 and 1655 cm^−1^. In PC2 loading plot, the bands at 721, 1002, 1156, 1340, 1526 cm^−1^ account for the maximum variance. To incorporate the significant Raman spectroscopy features, LDA was applied to generate a diagnostic algorithm using PC1 and PC2. Table 2 compares the histopathologic diagnoses with those of the Raman diagnostic algorithm for our data set. The algorithm results in a sensitivity for detecting ORMS of 91.3% (21/23), a specificity of 88.9% (24/27), and an overall accuracy of 90.0% (45/50).

**Table 2:** Comparison of histopathologic diagnosis with that of the Raman diagnostic algorithm.

Histopathologic diagnosis	Raman prediction		Total
	Rhabdomyosarcoma	Normal	
Rhabdomyosarcoma	21	2	23
Normal	3	24	27

## Discussion

Abnormal alterations in cellular structures and biochemical compositions in tumor stromal microenvironment lead to stipulated tumor growth. The various types of cells within tumor stroma have biochemical properties different from normal tissues. These subtle early changes generally would not cause obvious clinical symptoms and are often difficult to be detected by traditional medical imaging modalities. Raman spectroscopy is sensitive to the changes in molecular composition and conformation that occur during carcinogenesis, thus may contribute to objective, noninvasive, real-time diagnosis by way of an “optical biopsy” and a reduction of treatment delays. At present, Raman spectroscopy has been heralded as a most promising molecular tool for clinical diagnosis of cancer, including breast cancer^14^, gastric cancer^15^, colon cancer^16^, esophagus cancer^9^ and brain tumor^17^.

However, the research on Raman spectroscopy for diagnostics of rhabdomyosarcoma and orbital tumor is quite rare, except for two articles in the medical literature. Luther et al. reported a case of recurrent spheno-orbital meningioma extending into the middle cranial fossa, the lateral orbit, and the temporalis muscle^17^. Intraoperative stimulated Raman spectroscopy aided in the identification of tumor boundaries within the orbit and temporalis muscle in order to better preserve the important anatomic structures during resection surgery. In the other report, Kast et al. utilized Raman spectroscopy to differentiate neuroblastoma, rhabdomyosarcoma, Ewing sarcoma, and non-Hodgkin lymphoma in vitro, which share similar histological features. The study demonstrated that Raman spectroscopy is an accurate technique for quickly and accurately differentiating the above mentioned four types of small round blue cell tumors^18^. However, this research did not give detailed information about the biochemical changes in different tumor tissues.

In this work, we collect the Raman signatures of orbital tissues within the "molecular fingerprint" region in the wavenumber interval from 600 to 1800 cm^−1^. We also correlate the Raman spectra with representative histopathology of ORMS, showing elongated pleomorphic tumor cells with a centrally located hyperchromatic nucleus surrounded by a considerable amount of eosinophilic cytoplasm (Fig. 1A). While histopathology characterizations reveal the cellular and morphological features of ORMS, the Raman spectroscopy uncovers the unparalleled insights into the understanding of biochemical/biomolecular constituents of different orbital tissues. Figure 2 shows that the Raman spectra of ORMS and normal orbital tissue have good reproducibility, and the position of Raman peak of the two types of orbital tissues is roughly the same, indicating that the types of Raman-active molecules (such as nucleic acids, lipids, proteins, and carbohydrates) contained in the two different pathological types of orbital tissues are comparable. The main difference between the two spectra which can be viewed more clearly on the difference spectrum in Fig. 2 is the spectral peak intensity, reflecting alterations in the concentrations of a variety of biomolecules associated with malignant transformation. To better understand the molecular basis for the observed Raman spectra, Table 1 lists tentative assignments for the observed Raman bands, according to literature data. For instance, the prominent Raman peaks at 721, 1088 and 1340 cm^-1^ can be probably attributed to nucleic acids. The intensities of the above peaks are significantly higher in ORMS, revealing that the chromatin density related to the aggressive growth and abnormal differentiation of tumor cells is markedly higher in ORMS. We also found that Raman peaks at 758 and 1206 cm^-1^ assigned to tryptophan are significantly increased associated with ORMS, which is probably due to the increase of proliferation activity of ORMS. This observation is in agreement with the Raman molecular biology studies by Draga and Huang whereby an increase in the intensity of tryptophan peak associated with bladder cancer and lung cancer has been reported, respectively^19,20^. In addition, the Raman peak at 1002 cm^-1^ attributed to phenylalanine is also significantly stronger in ORMS. Phenylalanine has been considered as a biomarker of uncontrolled cell proliferation, which is widely present in processes of collagen fiber formation and malignant transformation^21^. Besides, the Raman peak intensities at 1156 and 1526 cm^-1^ assigned to carotenoid appeared to be more intense for ORMS than for normal tissue. Since carotenoid has been proved to be an effective antioxidant, this result provides strong evidence for the inference that ORMS acquires stronger ability to resist oxidative damage due to the increased content of carotenoid^12^. Of note, the normal orbital tissue showed the more intense band related to unsaturated fatty acids (1655 cm^−1^), corresponding to a large area of fatty tissue as demonstrated by the histopathology image (Fig. 1B).

To further improve the diagnostic performance of the fiber-optic Raman technique, we fully utilize all the diagnostically significant information contained in tissue Raman spectra by implementing sophisticated multivariate statistical analysis (PCA-LDA) for diagnostic model development. PCA is a data compression procedure which finds major trends within the spectral data set and redefines the data set using a small set of component spectra or PCs. Figure 4 demonstrated relatively good separation between the two groups, suggesting that there are inherent molecular differences between Raman spectra from ORMS and normal orbital tissue. LDA finds discriminant function that maximizes the variance in the data between groups and minimizes the variance between members of the same group. As shown in Table 2, the PCA-LDA based Raman biomolecular diagnostic algorithm yielded the diagnostic accuracy of 90.0% (i.e., sensitivity of 91.3% and specificity of 88.9%) for identifying ORMS from normal orbital tissue in vitro.

However, the sample size of our study was constrained by experimental conditions and maybe not large enough to explore more differences and relevant significance between ORMS and normal orbital tissue. So we will enlarge sample size to further investigate these changes in our future studies. Despite the small number of samples, the high predictive accuracy in this work reinforces the robustness of Raman spectroscopy for an optical diagnosis of ORMS at the molecular level. Future pragmatic developments could also aim to combine fiber optic Raman spectroscopy with other novel endoscopic imaging modalities and/or conventional medical imaging (eg, ultrasonography, CT, and MRI), which may be the directions to explore for functional imaging at the molecular, cellular, tissue, and even organ levels.

## Conclusions

In summary, we have acquired for the first time in vitro Raman spectra features from ORMS and normal orbital tissue. Distinctive spectral differences between the two types of tissues suggest the changes in the relative amounts of biochemical components, such as nucleic acids, tryptophan, phenylalanine, carotenoid and lipids. The PCA-LDA based algorithm is developed for providing good differentiation between normal and sarcoma tissues. Raman spectroscopy technique integrated with PCA-LDA multivariate analysis for efficient Raman features selection has potential for accurate, rapid and noninvasive optical diagnosis of ORMS at the molecular level.

### Supplementary Information

Supplementary Information 1.

Supplementary Information 2.

Supplementary Information 3.

Supplementary Information 4.

## Data Availability

Data is provided within the manuscript and supplementary information files.
