# Supplementary material for: Preliminary study on the optical diagnosis of orbital rhabdomyosarcoma by Raman spectroscopy
Source: Sci Rep. 2024 Apr 28;14:9735. doi: 10.1038/s41598-024-60520-w (PMC11056361; doi:10.1038/s41598-024-60520-w)
Supplement: Supplementary file 1 — Supplementary Information 1. [file 41598_2024_60520_MOESM1_ESM.docx]

Figure legend for supplementary Figure 1: Mean Raman spectra of orbital rhabdomyosarcoma (ORMS) tissue (blue line, n=23) and normal orbital tissue (red line, n=27) in vitro. ORMS minus normal orbital tissue is used to represent the mean difference spectrum (green line), which is shown at the bottom.
