# Supplementary figures and images for: Preliminary study on the optical diagnosis of orbital rhabdomyosarcoma by Raman spectroscopy
Source: Sci Rep. 2024 Apr 28;14:9735. doi: 10.1038/s41598-024-60520-w (PMC11056361; doi:10.1038/s41598-024-60520-w)

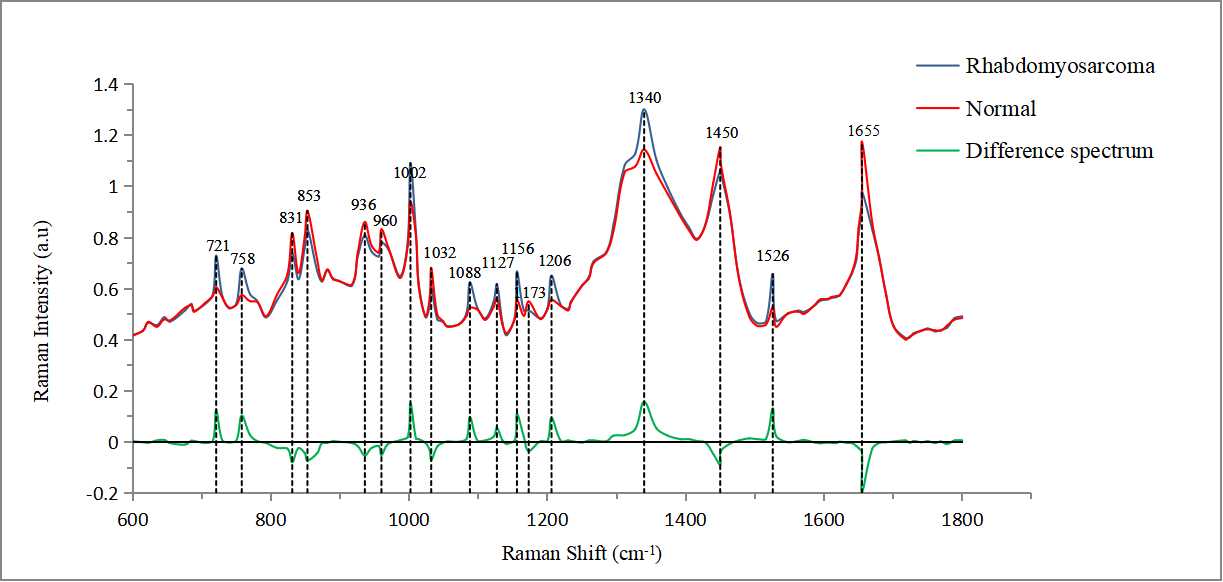

Supplement: Supplementary file 4 — Supplementary Information 4. [file 41598_2024_60520_MOESM4_ESM.jpg]
